# A Reciprocal Transplant Experiment Confirmed Mite-Resistance in a Honey Bee Population from Uruguay

**DOI:** 10.3390/vetsci9110596

**Published:** 2022-10-28

**Authors:** Yamandú Mendoza, Estela Santos, Sabrina Clavijo-Baquett, Ciro Invernizzi

**Affiliations:** 1 Sección Apicultura, Programa de Producción Familiar, Instituto Nacional de Investigación Agropecuaria La Estanzuela, Ruta 50 km 11, Colonia 70002, Uruguay; 2 Sección Etología, Facultad de Ciencias, Universidad de la República, Iguá 4225, Montevideo 11400, Uruguay; 3 Programa de Desarrollo de las Ciencias Básicas (PEDECIBA), Isidoro de María 1614, Montevideo 11800, Uruguay

**Keywords:** *Apis mellifera*, *Varroa destructor*, hygienic behavior, grooming, mite infestation, mite reproduction

## Abstract

**Simple Summary:**

In Uruguay, as in many countries around the world, the *Varroa destructor* mite is the main biotic threat to honey bees (*Apis mellifera*). Most beekeepers regularly apply acaricides to their colonies to have good honey harvests and avoid large losses, with the exception of beekeepers in the east of the country where bees coexist with V. destructor without suffering significant damage. To unravel the different *A. mellifera*–*V. destructor* relationships found in the country, a reciprocal transplant experiment was performed between the mite-resistant bee colonies and the mite-susceptible bee colonies from the east and the west of the country, respectively. The differences between the two groups of bees in the control of *V. destructor* were maintained in the two environments. No mite-susceptible colonies survived the winter. The behavioral resistance of bees (hygienic behavior) and reproductive aspects of *V. destructor* (phoretic mites/reproductive mites and mites in drone cells/mites in worker cells ratio) could explain the results obtained.

**Abstract:**

In the past few years there has been an increasing interest for the study of honey bee populations that are naturally resistant to the ectoparasitic mite *Varroa destructor*, aiming to identify the mechanisms that allow the bees to limit the reproduction of the mite. In eastern Uruguay there are still bees resistant to mites that survive without acaricides. In order to determine if the differential resistance to *V. destructor* was maintained in other environments, a reciprocal transplant experiment was performed between the mite-resistant bee colonies and the mite-susceptible bee colonies from the east and the west of the country, respectively, infesting bees with local mites. In both regions, the mite-resistant colonies expressed a higher hygienic behavior and presented a higher phoretic mites/reproductive mites and mites in drone cells/mites in worker cells ratio than the mite-susceptible colonies. All the mite-susceptible colonies died during fall–winter, while a considerable number of mite-resistant colonies survived until spring, especially in the east of the country. This study shows that the bees in the east of the country maintain in good measure the resistance to *V. destructor* in other regions and leaves open the possibility that the mites of the two populations have biases in the reproductive behavior.

## 1. Introduction

For over four decades, the mite *Varroa destructor* has constituted the main biotic threat to honey bees (*Apis mellifera*) in several countries throughout the world [1,2]. Besides the direct damage that *V. destructor* causes, mostly to the brood during the reproduction period, it also acts as a vector for several RNA viruses and favors their replication as it suppresses the immune response of the bees [3,4]. The most associated virus to *V. destructor* is Deformed Wing Virus (DMW) [5,6,7].

The relationship between honey bees and *V. destructor* varies according to the region of the world, being molded by natural selection, beekeeping practices, and artificial selection programs [8,9,10,11,12,13,14]. In many countries with advanced beekeeping industries, especially in those countries with a temperate climate, the damage that *V. destructor* causes forces beekeepers into the systematic use of acaricides in order to prevent major colony losses [2].

Honey bees present two behavioral resistance mechanisms to *V. destructor* that have been widely studied and included in genetic improvement programs. The hygienic behavior (uncapping of cells that contain dead, diseased, or parasitized brood and its subsequent removal) is a social behavior that helps control diseases in the brood such as the American foulbrood (causative agent *Paenibacillus larvae*) and the chalkbrood disease (causative agent *Ascosphaera apis*), and can interrupt the reproduction of *V. destructor* [15]. Several studies have found that bee colonies with a high hygienic behavior have a better control of the *V. destructor* population [16,17,18,19,20,21,22,23], though others did not find this association [24,25]. In the USA, *V. destructor*-resistant colonies have been selected; their main characteristic is the ability that bees have to detect and clean cells with pupa parasitized by the mite, naming this characteristic VSH (Varroa Sensitive Hygiene) [20,26,27]. Grooming behavior, by which parasitized bees can dislodge mites by themselves (autogrooming) or receiving help from other bees (allogrooming) [28], has been reported as an effective resistance mechanism against *V. destructor* [24,29,30,31], although other studies have not found an association between this behavior and the prevalence of the mite [32,33,34]. Both the grooming and hygienic behavior are expressed in a very efficient way in the Asian honey bees *Apis cerana*, the natural host of *V. destructor*, and would be keys to control the parasite’s population [35,36,37]. In *A. mellifera* both behaviors are expressed better in Africanized bees (*A. m. scutellata* hybrids) than in bees from European subspecies, which may explain in part the known resistance to *V. destructor* that Africanized bees present in Brazil [9].

Over the last few years, studies on bee populations that naturally survive *V. destructor* have gained relevance [9,12]. These studies have shown that natural selection has favored several resistance mechanisms in bee populations, including different components of the behavioral resistance of bees, limitation of mite reproduction, and the reduction of the colonies’ sizes [9,12]. The usual reference to bee populations that are “resistant” or “survivors” to *V. destructor* does not mean that they have total resistance to the mite, but rather a significantly lower probability to collapse due to the parasitosis than most bee populations.

In Uruguay, beekeepers must apply acaricides regularly in order to prevent major losses during fall and winter. However, in the east of the country there are still bee populations who present a good resistance to *V. destructor* and survive very well without acaricides. Recently Mendoza et al. [38] performed an exhaustive comparison between a mite-resistant population of bees from the east coast and a mite-susceptible from the west coast in order to determine the factors that influence a different relationship between the host and the parasite. They found that mite-resistant colonies presented a higher hygienic and grooming behavior than mite-susceptible colonies. At the end of the summer, the mite-resistant colonies had fewer mites and a lower DWV (type A) infection intensity than the mite-susceptible colonies. A molecular analysis (microsatellites) showed that mite-resistant bees were Africanized (*A. mellifera scutellata* hybrids) while the mite-susceptible bees were closer to European subspecies. An interesting result of this study was that mite-resistant colonies presented both the phoretic mites/reproductive mites ratio and the mites in drone cells/mites in worker cells ratio in a higher level than the mite-susceptible colonies. These differences may be due to differences between mites since genetic differences (microsatellites) were found in both populations. The death during fall of all the mite-susceptible colonies, but only 18% of the mite-resistant colonies clearly showed the differences between the two *A. mellifera*–*V. destructor* systems studied. This way, it cannot be discarded that the differences in the survival of the colonies of the two analyzed populations in Mendoza et al. [38]’s study are due to the differences in the reproductive behavior of the mites in each region.

The aim of this study was to compare the behavioral resistance of bees to *V. destructor*, the evolution of the infestation, and the reproductive aspects of mites in apiaries with mite-susceptible and mite-resistant colonies located in two regions, one where mite-susceptible bee populations predominate and one where mite-resistant bee populations predominate. 

## 2. Material and Methods

### 2.1. Overview

During the month of November (spring in the southern hemisphere), bee nucs were formed in the experimental station INIA La Estanzuela (Apiary S, 34°20′48″ S; 57°41′29″ O, in the western region of the country, where mite-susceptible bees predominate) and in the experimental station INIA Treinta y Tres (Apiary R, 33°15′06″ S; 54°25′40″ O, in the eastern region of the country, where mite-resistant bees predominate). These were the apiaries used in the study performed by Mendoza et al. [38]. In each nuc, a local queen obtained from the apiary itself was introduced. During December, some of the mite-susceptible colonies were transported from Apiary S to Apiary R and some of the mite-resistant colonies from Apiary R to Apiary S. Before the transportation, the colonies were treated with an acaricide (flumethrin). Once they were installed in their new apiaries, the colonies received capped brood frames from the other colonies of the apiary (kept uncured) in order to be infested by local mites. No colony received an acaricide subsequently. During summer, Apiary S was formed by 20 mite-susceptible colonies and 18 mite-resistant colonies, while Apiary R was formed by 19 mite-susceptible colonies and 20 mite-resistant colonies. Langstroth hives were used in both apiaries.

### 2.2. Estimation of the Bee Population and Brood Area

Between the months of February and October, the colonies from both apiaries were inspected 7 times, estimating the bee population (number of combs covered by bees) and brood area (faces of combs with brood) [39]. 

### 2.3. Evaluation of Hygienic and Grooming Behaviors 

In February (Apiary S) and March (Apiary R), the hygienic behavior of the colonies was evaluated. At least 100 pupae were killed by piercing them with an entomological pin through the cell cap and 24 h later the number of removed pupae was recorded. The result was expressed as a percentage of cleaned cells [40]. 

In April (Apiary S) and May (Apiary R), grooming behavior of the colonies was evaluated. A petroleum jelly-smeared sheet was placed on the floor of the hives for 7 days so that the mites dislodged by the bees would remain attached. Mites were observed at 40× under microscope to determine if they had mutilated legs. Grooming behavior was expressed as a percentage of damaged mites [41].

### 2.4. Estimation of Mites on Bees and in Brood Cells 

During the same days that adult population and brood area were estimated, the *V. destructor* infestation on adults bees was evaluated. For this, a sample of approximately 300 workers collected in three brood combs was taken from each colony. The mites were dislodged from the bees with 75% ethanol, and the percentage of infested bees was calculated [42].

Furthermore, during February (Apiary S) and April (Apiary R), on the occasion of the second and third register of phoretic mites respectively, the number of adult mites (female founders) and their offspring were recorded in 400 worker cells. From these data, the fertility of the mites (cells infested with one mite with offspring), the abundance (the average number of adult female mites per examined cells), the intensity (the average number of female mites per infested cell), and the prevalence (the percentage of infested cells) were estimated. The relation between the infestation level in adult bees and brood (abundance) was determined.

In those colonies that still had drone brood (over 20 cells), the cells were uncapped in order to determine the presence of mites and study the relation between the mites present in the drone cells and the mites present in the worker cells. Thus, 6 mite-susceptible colonies and 14 mite-resistant colonies in Apiary S and 14 mite-susceptible colonies and 7 mite-resistant colonies in Apiary R were analyzed.

### 2.5. Statistical Analysis

Prior to comparing the different variables between the mite-susceptible and mite-resistant colonies in each apiary, the data were analyzed in order to determine whether or not they followed a normal distribution (Shapiro–Wilk test) and presented homogeneity of variance (Bartlett test) to be able to apply parametric tests. Not having met these conditions, non-parametric tests had to be used. 

Wilcoxon test was used in order to compare the adult population, brood area, hygienic behavior, grooming behavior, level of infestation by *V. destructor* in adult bees, fertility, abundance, prevalence and intensity of *V. destructor* in brood cells, and the phoretic mites/reproductive mites ratio (adding 1 to the numerator and denominator to avoid having values in 0) between the mite-susceptible and mite-resistant colonies.

The mortality of the colonies in both groups throughout the overwintering in both apiaries was compared using the proportions test. 

Infestation of *V. destructor* in drone and worker cells on mite-susceptible and mite-resistant colonies from both apiaries was analyzed performing generalized linear models (GLMs) with a logit function. The response variable was presence (1) or absence (0) of mites and the predictor variables were cell type (i.e., worker or drone cells), honey bee population (mite-susceptible and mite-resistant), apiary (Apiary S and Apiary R), and interaction terms among them. Model selection was carried out with an Akaike information criterion (AIC) [43,44], and the best fit for the data was achieved for the model with the lowest AIC value (∆AIC > 2). All the mentioned analyses were performed using the statistic program R [45]. Values of P under 0.05 were considered as statistically significant differences.

## 3. Results

### 3.1. Evolution of Colonies Strength

The mite-susceptible and mite-resistant colonies installed in Apiary S showed at the beginning of February the same adult and brood population. However, by the end of February and until midway through June, the mite-resistant colonies were larger than the mite-susceptible local colonies (Table 1). The mortality of the colonies between groups during fall and winter also presented notable differences. While by August, 100% of the local mite-susceptible colonies were dead, the mite-resistant colonies made it to Spring with 61% losses (Table 1).

The mite-susceptible and mite-resistant colonies installed in Apiary R showed at the beginning of February the same population, but the brood area was 20% less in mite-resistant colonies. During March and April, the colonies from both groups were the same size but in May and June, the mite-resistant colonies had a larger amount of bees than the mite-susceptible colonies (Table 1). The mortality of both groups also presented notable differences. By June, 75% of the mite-susceptible colonies had died, and by August, the mortality had reached all the colonies; the mite-resistant colonies only had a 35% loss during the overwintering (Table 1).

### 3.2. Hygienic and Grooming Behavior

**Figure 1:**
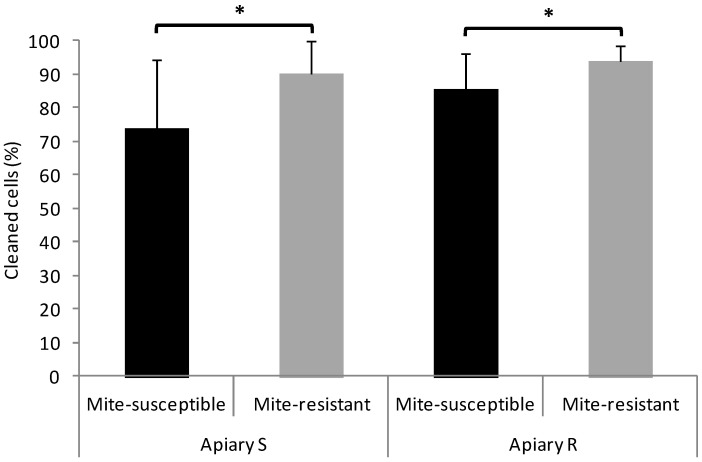

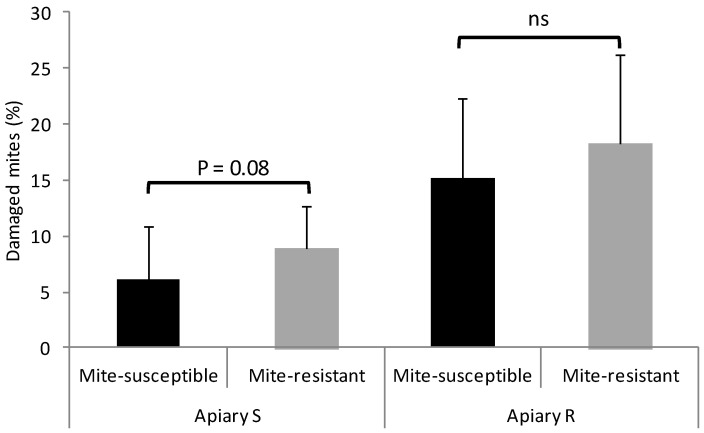
Hygienic and grooming behaviors of the mite-susceptible and mite-resistant colonies in the apiaries with local mite-susceptible bee population (Apiary S) and mite-resistant bees (Apiary R). The * indicates significant differences (*p* < 0.05) for the Wilcoxon test; ns: non significant.

In the two apiaries, mite-resistant colonies showed more hygienic and grooming behavior than the mite-susceptible colonies, although for the latter behavior the statistical difference was marginally significant in Apiary S and non-significant in Apiary R (Figure 1).

### 3.3. Estimation of Mites on Bees and in Brood Cells

**Figure 2:**
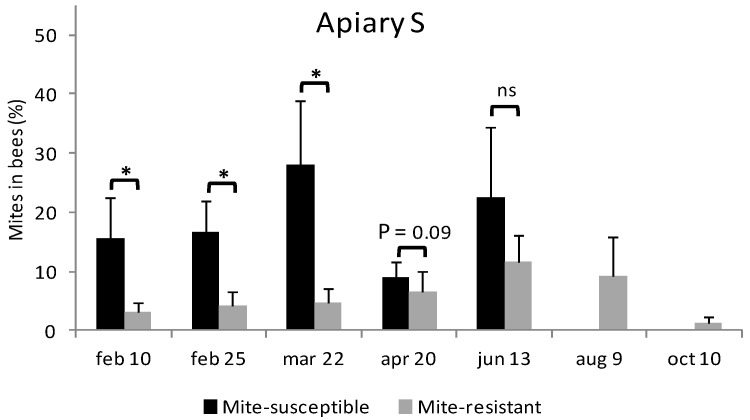

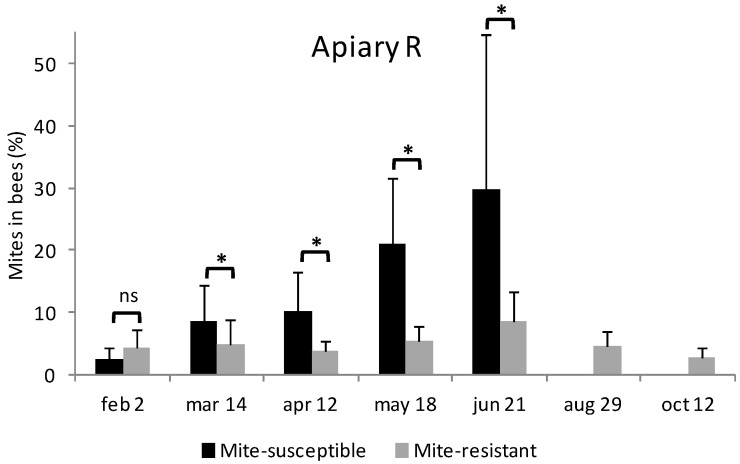
Evolution of the level of infestation by *V. destructor* in mite-resistant and mite-susceptible colonies in the apiaries with local mite-susceptible bees (Apiary S) and mite-resistant bees (Apiary R). The * indicates significant differences (*p* < 0.05) for the Wilcoxon test; ns: non significant.

In Apiary S during February, the mite-susceptible colonies were more infested by *V. destructor* than the mite-resistant colonies (15.5 ± 7.0% y 3.1 ± 1.6%, respectively) (Figure 2). This difference on the level of infestation was on the rise until 22 March. On 20 April no differences in the level of infestation were found, although by this date 60% of the mite-susceptible colonies had died (Table 1). In the last two samplings there were no surviving mite-susceptible colonies. 13 June was the biggest infestation moment for mite-resistant colonies, from which it started to decrease (Figure 2).

In Apiary R during February, the mite-resistant colonies were more infested by *V. destructor* than the mite-susceptible colonies (4.4 ± 3.0% y 2.5 ± 1.8%, respectively) (Figure 2). However, on March 14th the mite-resistant colonies were less infested than the mite-susceptible colonies (5.0 ± 3.9% y 8.7 ± 5.7%, respectively) (Figure 2). This difference began increasing through the fall. The level of infestation by *V. destructor* in the mite-susceptible colonies reached its peak value on June 21st. In the following inspection to the apiary no mite-susceptible colonies were found alive. Mite-resistant colonies also reached their infestation peak on June 21st from which date the infestation decreased (Figure 2).

The presence of mites in the brood was registered once in the colonies. In both apiaries mite-susceptible and mite-resistant colonies did not show differences in the fertility of *V. destructor*. In Apiary S the proportion of fertile mites in mite-susceptible colonies was 86.2% ± 12.2% and in mite-resistant colonies 85.3% ± 18.6% (W = 149.5; *p* = 0.713). In Apiary R the proportion of fertile mites in mite-susceptible colonies was 86.3% ± 14.8% and in mite-resistant colonies 86.6% ± 21.4% (W = 133; *p* = 0.499). 

**Figure 3:**
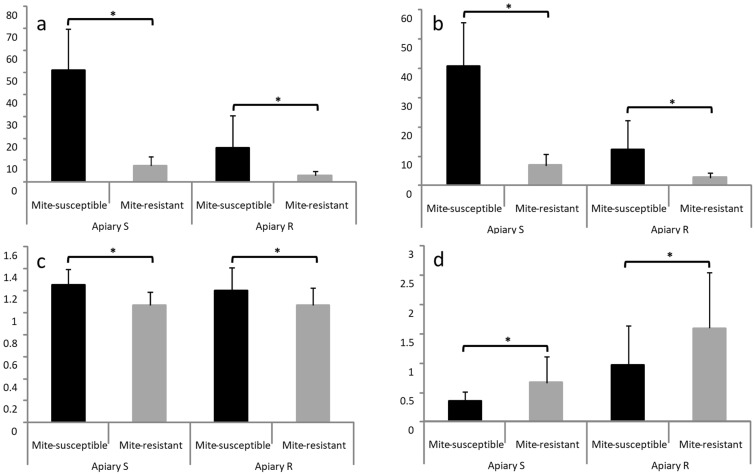
Presence of *V. destructor* in the mite-susceptible and mite-resistant colonies brood in the apiaries with local mite-susceptible bees (Apiary S) and mite-resistant bees (Apiary R). Abundance in brood cells (**a**), prevalence in brood cells (**b**), intensity of infestation in brood cells (**c**), and relationship between phoretic and reproductive mites (**d**). The * indicates significant differences (*p* < 0.05) for the Wilcoxon test.

In both apiaries, abundance, intensity, and prevalence of *V. destructor* in brood cells were less in mite-resistant colonies than in mite-susceptible colonies (Figure 3). 

The phoretic mites/reproductive mites ratio was estimated dividing the percentage of mites on bees (corresponding to the logging date of the presence of parasite in the brood) by the percentage of mites in worker bee cells (abundance). This relation was higher in mite-resistant colonies than in mite-susceptible colonies in both apiaries (Figure 3). 

Table 2 presents the values of abundance, intensity, and prevalence of *V. destructor* in the total of drone and worker cells inspected in mite-susceptible and mite-resistant colonies in Apiary S and Apiary R. In general terms, in both apiaries mite-resistant colonies had fewer mites in drone and worker cells than the mite susceptible colonies. Regarding the mites in drone cells/mites in worker cells ratio, it was higher in mite-resistant colonies than in mite-susceptible colonies, especially in Apiary R. It is also verified that this relation is higher for both groups of colonies in Apiary R than in Apiary S. 

**Figure 4:**
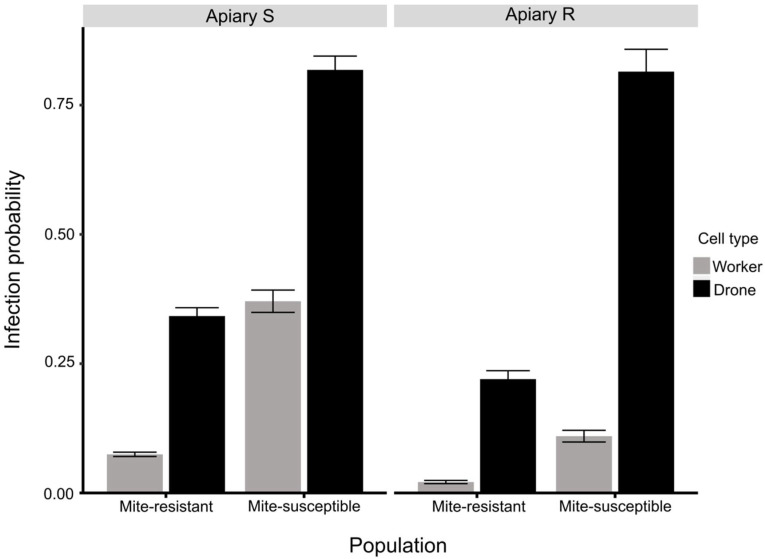
Estimated probability of *V. destructor* infestation in drone and worker cells in mite-resistant and mite-susceptible colonies in the apiaries with original population of mite-susceptible bees (Apiary S) and mite-resistant bees (Apiary R) according to the generalized linear model (GLM) analysis with link logit.

The best fitted model for *V. destructor* infestation included as predictor variables apiary, honey bee population, cell type, and interaction terms among them (Figure 4, Tables S1 and S2). Cells from colonies of Apiary R experienced 74% less infestation than cells from colonies of Apiary S (Odd-ratio = 0.27). Besides, drone cells were six times more likely to be infested than worker cells (Odd-ratio = 6.45). Mite-susceptible colonies were seven times more likely to be infested than mite-resistant colonies (Odd-ratio = 7). Drone cells infestation from mite-susceptible and mite-resistant populations were not significantly different between apiaries (Odd-ratio = 1). Nevertheless, in Apiary S drone cells from mite-resistant colonies were four times less likely to be infested (Odd-ratio = 0.52) than cells drones from mite-susceptible (Odd-ratio = 4.51), though Apiary R drone cells from mite-resistant colonies had the same likelihood to be infested (Odd-ratio = 0.28) as cells drones from mite-susceptible (Odd-ratio = 4.40). Worker cells from mite-resistant colonies were more infested in the Apiary S (Odd-ratio = 0.158) than Apiary R (Odd-ratio = 0.589). In Apiary R worker cells from mite-resistant colonies were 14% less likely to be infested (Odd-ratio = 0.16) than worker cells from mite-susceptible colonies (Odd-ratio = 0.59). In Apiary S worker cells from mite-resistant colonies were 50% less likely to be infested (Odd-ratio = 0.08) than worker cells from mite-susceptible colonies (Odd-ratio = 0.59).

## 4. Discussion

The study of honey bee populations that naturally survive *V. destructor* allows us to unravel the relationship between these two organisms and eventually count on new tools to control the parasite. Recently, Mendoza et al. [38] described the relationship between honey bees and *V. destructor* in two distant regions of Uruguay with different consequences regarding the survival of the colonies. In this study, differences in the reproductive behavior and genetics differences between the mites of both regions were found, which could partly explain the results. In order to shed light in this aspect, mite-susceptible and mite-resistant colonies of the two apiaries used by Mendoza et al. [38] were interchanged and the response of both types of colonies facing the local mites was analyzed. 

The survival of the mite-susceptible and mite-resistant colonies in both apiaries showed marked differences. While 100% of the mite-susceptible colonies died before spring in both apiaries, only 61% and 35% of the mite-resistant colonies died in Apiary S and Apiary R, respectively. These mortality values resemble those found by Mendoza et al. [38] where the totality of mite-susceptible colonies did not make it through the winter, while only 18% of the mite-resistant colonies died during this period in their apiaries of origin. The loss of mite-resistant colonies was high on Apiary S. However, the survival of 40% of the mite-resistant colonies in this apiary is remarkable considering they were on a region with a high density of colonies where beekeepers apply acaricides regularly to prevent massive losses throughout the year [38]. 

In each apiary, the loss of colonies of each group was preceded by differences in the strength of the colonies, after starting in similar conditions. In Apiary S, near the end of March, 40 days after the start of the study, local mite-susceptible colonies presented on average half of the population of bees compared with the mite-resistant ones. The following month, 60% of mite-susceptible colonies died. In Apiary R the same trend repeated itself, but not as pronounced. In May, mite-susceptible colonies presented in average a little more than half of the population compared with mite-resistant colonies. The following month, 74% of the mite-susceptible colonies died. This way, in both apiaries it was verified that colonies susceptible to *V. destructor* suffered from a rapid depopulation that ended in their collapse. Rosenkranz et al. [2] indicated that *V. destructor* causes depopulations in the colonies that end on their deaths, following a picture named “Parasitic mite syndrome”, characterized by the presence of scattered brood and loss of adult bees. 

The increase in the *V. destructor* population in mite-susceptible and mite-resistant colonies was clearly different in both apiaries. Mite-susceptible colonies could not avoid a fast increase in the level of infestation that reached on average 30% before they collapsed. Even in Apiary R, where the average level of initial infestation was low (2.5%), mite-susceptible colonies died at the beginning of winter. On the other hand, in mite-resistant colonies, the increase of the mite population was more controlled in both apiaries, recording fewer than 10% in average in most of the fall and winter months and with a decrease at the beginning of spring. The ability of the mite-resistant colonies to limit the growth of the mite population suggests these bees effectively present resistance to the parasite (limitation of its reproduction) and no tolerance to it (attenuation of its effects) [12]. The rate of increase in the mite population is a characteristic that has been taken into account as a trait to select in order to obtain resistant colonies [46,47]. 

Mendoza et al. [38], when analyzing the same bee populations of this study, found that mite-resistant colonies presented a higher hygienic and grooming behavior than mite-susceptible colonies, noting that the behavioral resistance could explain a good part of the difference between both groups of colonies in controlling *V. destructor*. At the time of evaluating these two behaviors in mite-susceptible and mite-resistant colonies in each apiary, it was found that in general terms the differences remained, especially regarding the hygienic behavior (only in Apiary S marginal differences were found in the grooming behavior). These results rule out that the differences found by Mendoza et al. [38] when evaluating the colonies in their place of origin were due to environmental effects. The importance of hygienic behavior measured as the response of the bees facing the killed brood by freezing or puncturing, in the resistance to *V. destructor* has yet to reach consensus [16,17,18,19,20,21,22,23,24,25]. The results of this study, added to those obtained by Mendoza et al. [38], would show that the hygienic behavior of mite-resistant bees in Uruguay is a characteristic associated with control over the parasite. An aspect to elucidate in the future is whether mite-resistant colonies with a high hygienic behavior also possess the VSH trait, a specialization of the hygienic behavior aimed to detect pupae parasitized by *V. destructor* [20,26,27]. The association between these two characteristics is still debatable, for example, it is possible that the stimuli that trigger the cleaning response are different [12,15]. Regarding grooming behavior, its contribution to the control over *V. destructor* in the mite-resistant colonies analyzed is less clear and requires more studies. 

When analyzing the presence of *V. destructor* in the brood it was found that the fertility of the mite did not present differences in the colonies of both groups in neither of the apiaries, with values that oscillated between 85.3% and 86.6%, similar to those found by Mendoza et al. [38] when the colonies were assessed in their place of origin. This confirms that the fertility of *V. destructor* is not a factor that explains the differential damage of the mite in the two bee populations. However, other studies in populations of bees naturally surviving *V. destructor* attribute its reduced impact on the colonies to the low fertility of the mite [24,48,49,50]. On the other hand, differences in the abundance, intensity, and prevalence of *V. destructor* were found in the colonies of the two groups in both apiaries. These differences were expected and follow the different level of infestation in the two groups of colonies at the moment of the evaluation. Mite-susceptible colonies had more mites on the bees than mite-resistant colonies in both groups (four times more in Apiary S and two times more in Apiary R). 

When analyzing the phoretic mites/reproductive mites ratio it was found that in both apiaries this relation was higher in mite-resistant colonies than in mite-susceptible colonies (two times more in Apiary S and one and half times more in Apiary R). In any case, it is remarkable that beyond the differences found in this ratio between the two groups of colonies, the average value of Apiary S was less than half of the one found in Apiary R. Mendoza et al. [38] also found differences in the same sense in the phoretic mites/reproductive mites ratio between mite-resistant and mite-susceptible colonies (four times more) when assessed in their place of origin. The authors pointed out that the differences could be explained by the better hygienic behavior presented in mite-resistant colonies that interrupts the reproduction cycle *V. destructor*, eventually eliminating them or forcing them to enter phoresis which reduces the chance of reproducing successfully [51,52]. Oddie et al. [53] found in bee populations that naturally survive *V. destructor* in Europe that bees have a higher tendency to uncap the infested cells and later recap them compared with the local mite-susceptible bees. The authors suggest that this behavioral response, which reduces the cost of removing pupae, was acquired by these bee populations through a rapid selection process (but see ref. [54] for another interpretation). In this line of thought, Martín et al. [55] recently found that the recapping of parasitized cells is expressed more in bees resistant to *V. destructor* than in bees from places where mites are still not present (“naive bees”). The recapping behavior and its possible consequence in the phoretic mites/reproductive mites ratio is an aspect to study in mite-susceptible and mite-resistant bee populations. 

When analyzing the mites in drone cells/mites in worker cells ratio the mite-resistant colonies presented values slightly higher than the mite-susceptible colonies in Apiary S (6.5:1 y 5.0:1, respectively) and higher in Apiary R (16.9:1 y 12.1:1, respectively). The generalized linear model (GLM) showed *V. destructor* infestation probability values in cells of drones and workers of mite-susceptible and mite-resistant colonies in the two apiaries consistent with these results. Mendoza et al. [38] found similar differences (5.7:1 in Apiary S y 12.6:1 in Apiary R) when assessing the colonies of each group in their place of origin. The differences in the preference of the mites for reproducing in drone cells or worker cells found in the two studies could be affected by the difference in the population of mites of each region. In support of this conjecture, Mendoza et al. [38] detected genetic differences between the two populations using molecular analysis (microsatellites). An increase in the reproduction of *V. destructor* in worker cells would lead to a lower bee replacement due to the elimination of parasitized larvae. Moreover, the larvae parasitized by *V. destructor* lead to bees with a lower life expectancy [56,57], reduce the immune response [3], and increase the viral load of DWV [5,58]. Ultimately, the viability of the colonies and their chances of reproduction would be reduced.

Striking differences appear when comparing at a global level the results found in the apiaries R and S, both in the phoretic mites/reproductive mites ratio, as well as in the mites in drone cells/mites in worker cells ratio. These differences indicate that mites from both apiaries could have a different behavior that affects the relationship they have with honey bees. The genetic differences between the mites from both apiaries found by Mendoza et al. [38] and morphological differences previously found between populations of mites from the east and west of the country [59] sustain the possibility that the populations on both regions are under different selection pressures that affect their reproductive behavior, ultimately affecting the colonies in different ways (but see ref. [60] for a different result). In that sense, it is to be noted that in the region where Apiary S is set there is a profuse beekeeping activity, with a colony concentration 10 times higher than in the region where Apiary R is set. The intense beekeeping activity could favor the increase in the virulence of *V. destructor* [11,61]. Aspects from the reproductive behavior of *V. destructor* could be studied in the future, for instance, by analyzing the preferences to parasite drone or worker cells in controlled laboratory conditions following established protocols [42]. 

Finally, it must be considered that the difference in resistance to *V. destructor* between the two populations of bees may be associated with the genetic origin. Mendoza et al. [38] found that mite-resistant bees were hybrids of the African subspecies *A. m. scutellata* and mite-susceptible bees were closer to European subspecies. Africanized bees generally show resistance to *V. destructor*, highlighting their hygienic and grooming behavior [9].

## 5. Conclusions

This study confirms the existence of a population of mite-resistant honey bees in Uruguay and proposes that the hygienic behavior, possibly associated with the Africanization of these bees, contributes to the control of the parasite. It also suggests that some reproductive aspects *V. destructor* should be considered while analyzing the effect of parasitosis in the colonies.

## Figures and Tables

**Table 1:** Population (number of combs covered by bees), brood area (faces of combs with brood), and mortality (%) in mite-susceptible (M-S) and mite-resistant (M-R) colonies in the apiaries with local mite-susceptible bees (Apiary S) and mite-resistant bees (Apiary R). Different letters for the variables population and brood indicate significant differences (*p* < 0.05) for the Wilcoxon test and different letters for the mortality variable indicate significant differences (*p* < 0.05) for the proportions test.

Apiary S								
Date		10 Feb.	25 Feb.	22 Mar.	20 Apr.	13 Jun.	9 Aug.	10 Oct.
Population	M-S	8.9 ± 1.7 ^a^	7.7 ± 1.3 ^a^	4.0 ± 1.8 ^a^	4.1 ± 1.6 ^a^	1.8 ± 1.3 ^a^	-	-
	M-R	9.3 ± 2.2 ^a^	9.4 ± 1.7 ^b^	9.5 ± 1.9 ^b^	9.2 ± 1.8 ^b^	5.7 ± 2.0 ^b^	2.4 ± 1.3	3.9 ± 2.2
Brood	M-S	4.3 ± 0.7 ^a^	3.8 ± 1.6 ^a^	2.5 ± 1.5 ^a^	2.2 ± 0.9 ^a^	0.1 ± 0.1 ^a^	-	-
	M-R	5.0 ± 1.4 ^a^	5.3 ± 1.1 ^b^	4.4 ± 1.4 ^b^	2.2 ± 0.9 ^a^	0.5 ± 0.5 ^b^	0.5 ± 0.3	2.3 ± 1.5
Mortality (%)	M-S	0 ^a^	0 ^a^	10 ^a^	60 ^a^	75 ^a^	100 ^a^	100 ^a^
	M-R	0 ^a^	0 ^a^	0 ^a^	0 ^b^	0 ^b^	28 ^b^	61 ^b^
Apiary R								
Date		2 Fe b.	14 Mar.	12 Apr.	18 May.	21 Jun.	29 Aug.	12 Oct.
Population	M-S	9.6 ± 1.7 ^a^	6.7 ± 1.9 ^a^	7.4 ± 2.8 ^a^	5.1 ± 2.6 ^a^	3.6 ± 1.7 ^a^	-	-
	M-R	9.7 ± 2.1 ^a^	7.3 ± 1.3 ^a^	9.0 ± 1.1 ^a^	8.4 ± 1.9 ^b^	6.1 ± 2.1 ^b^	6.1 ± 2.5	8.6 ± 3.0
Brood	M-S	5.4 ± 1.3 ^a^	4.3 ± 1.7 ^a^	3.2 ± 1.8 ^a^	1.8 ± 1.6 ^a^	1.5 ± 1.3 ^a^	-	-
	M-R	4.3 ± 0.9 ^b^	3.7 ± 0.7 ^a^	3.8 ± 1.1 ^a^	1.6 ± 0.6 ^a^	1.0 ± 0.6 ^a^	2.6 ± 1.2	4.2 ± 1.9
Mortality (%)	M-S	0 ^a^	0 ^a^	5 ^a^	21 ^a^	74 ^a^	100 ^a^	100 ^a^
	M-R	0 ^a^	5 ^a^	10 ^a^	10 ^a^	10 ^b^	15 ^b^	35 ^b^

**Table 2:** Indicators of the level of infestation by *V. destructor* in the totality of inspected drone cells and worker cells, as well as the relationship between the mites found in drone cells and workers cells (abundance) in mite-susceptible and mite-resistant colonies in the apiaries with original population of mite-susceptible bees (Apiary S) and mite-resistant bees (Apiary R). The parentheses indicate the number of colonies used.

		Colonies	Mite-Susceptible	Mite-Resistant
**Apiary S**	Drones	Inspected cells	209 (6)	871 (14)
		Mite-infested cells	171	298
		Total mites	497	468
		Abundance	2.4	0.5
		Intensity	2.9	1.6
		Prevalence (%)	81.8	34.2
	Workers	Inspected cells	499 (6)	4005 (14)
		Mite-infested cells	185	299
		Total mites	237	327
		Abundance	0.5	0.1
		Intensity	1.28	1.09
		Prevalence (%)	37.1	7.5
		Mites in drone/worker cells	5,0	6,5
**Apiary R**	Drones	Inspected cells	81 (3)	654 (7)
		Mite-infested cells	66	144
		Total mites	143	239
		Abundance	1.8	0.4
		Intensity	2.17	1.66
		Prevalence (%)	81.5	22.0
	Workers	Inspected cells	765 (3)	2130 (7)
		Mite-infested cells	84	45
		Total mites	112	46
		Abundance	0.1	0.02
		Intensity	1.33	1.02
		Prevalence (%)	11.0	2.1
		Mites in drone/worker cells	12.1	16.9

## Supplementary Materials

The following supporting information can be downloaded at: https://www.mdpi.com/article/10.3390/vetsci9110596/s1, Table S1: Model selection for Varroa destructor through AIC and log ratio test (LRT): Table S2: The best fitted model for Varroa destructor infestation.
