# Supplementary material for: A Reciprocal Transplant Experiment Confirmed Mite-Resistance in a Honey Bee Population from Uruguay
Source: Vet Sci. 2022 Oct 28;9(11):596. doi: 10.3390/vetsci9110596 (PMC9694040; doi:10.3390/vetsci9110596)
Supplement: Supplementary file 1 [file vetsci-09-00596-s001.zip › Table S1.pdf]

Table S1. Model selection for *Varroa destructor* infestation according to apiary and mite-resistance of the bee population through AIC and log ratio test (LRT).

Model	df	AIC	Deviance	LRT	p
A + C + P + AxP + PxC + AxC + AxPxC	8	5851.04		−2917.5	
A + C + P + AxP + PxC + AxC	7	5853.07	−4.03	−2919.5	0.0446
A + C + P + AxP + PxC	6	5878.95	−27.88	−2933.5	0.0000
A + C + P + AxP	5	5886.56	−9.61	−2938.3	0.0019
A + C + P	4	5890.89	−6.34	−2941.4	0.0118
A + P	3	6822.89	−934.00	−3408.4	0.0000
A + C	3	6496.24	−607.35	−3245.1	0.0000
P + C	3	6101.64	−212.751	3047.8	0.0000
P	2	7032.82	−211.93	−3514.4	0.0000
A	2	7359.95	−539.06	−3678.0	0.0000
C	2	6631.20	−136.96	−3313.6	0.0000

A: Apiary with two levels, original population of mite-susceptible bees (Apiary S) or mite-resistant bees (Apiary R); C: Cell type with two levels, drone cells or worker cells; P: Mite-resistance bee population with two levels, mite-susceptible or mite-resistant bees.
