# Supplementary material for: A Reciprocal Transplant Experiment Confirmed Mite-Resistance in a Honey Bee Population from Uruguay
Source: Vet Sci. 2022 Oct 28;9(11):596. doi: 10.3390/vetsci9110596 (PMC9694040; doi:10.3390/vetsci9110596)
Supplement: Supplementary file 1 [file vetsci-09-00596-s001.zip › Table S2.pdf]

Table S2. The best fitted model for *Varroa destructor* infestation.

Predictors	Odds Ratios	CI	Estimate	Std. Error	p
(Intercept)	0.08	0.07–0.09	−2.517	0.06	< 0.001
A (R)	0.27	0.19–0.37	−1.319	0.16	< 0.001
P (Mite-susceptible)	7.30	5.88–9.07	1.988	0.11	< 0.001
C (Drone)	6.45	5.37–7.74	1.865	0.09	< 0.001
A (R) X P (Mite-susceptible)	0.78	0.51–1.20	−0.245	0.22	0.265
A (R) X C (Drone)	2.03	1.37–3.01	0.708	0.20	< 0.001
P (Mite-susceptible) X C (Drone)	1.18	0.77–1.83	0.170	0.22	0.446
A (R) X P (Mite-susceptible) X C (Drone)	2.30	1.01–5.24	0.833	0.42	< 0.05
Explained Deviance / Nagelkerke's R^2			0.219/0.293		

A: Apiary with two levels, original population of mite-susceptible bees (Apiary S) or mite-resistant bees (Apiary R); C: Cell type with two levels, drone cells or worker cells; P: Mite-resistance bee population with two levels, mite-susceptible or mite-resistant bees.
